# Chronic Severe Aortic Regurgitation in a Child Due to Congenital Hypoplastic Right Coronary Cusp: A Report of a Rare Case

**DOI:** 10.7759/cureus.96460

**Published:** 2025-11-09

**Authors:** Avinash D Arke, Ajay Pandey, Rinita Ajey, Priyanka Balkhe, Ravindra Varma

**Affiliations:** 1 Cardiology, Jagjivanram Hospital, Mumbai, IND; 2 Cardiothoracic Surgery, Jagjivanram Hospital, Mumbai, IND

**Keywords:** aortic valve anomaly, congenital heart disease, hypoplastic cusp, right coronary cusp, valvular heart disease

## Abstract

Congenital hypoplasia of the aortic valve cusp is extremely rare. Such anomalies may predispose to valvular dysfunction, endocarditis, and, in some cases, sudden cardiac events.

We report a very rare case of a 13-year-old girl presenting with failure to thrive and exertional dyspnea. Clinical examination revealed a grade 3/6 to-and-fro ejection systolic and early diastolic murmur at the right upper sternal border. Transthoracic echocardiography revealed a tricuspid aortic valve with a markedly hypoplastic right coronary cusp, leading to severe eccentric aortic regurgitation. CT angiography confirmed asymmetric cusp morphology. The patient underwent surgical aortic valve replacement with a mechanical prosthesis. Postoperative recovery was uneventful.

Hypoplastic right coronary cusp is a rare anomaly of the aortic valve. Early recognition using multimodality imaging and timely surgical intervention are essential for preventing adverse outcomes.

## Introduction

Congenital abnormalities of the aortic valve are frequently encountered in the form of a bicuspid aortic valve, with a prevalence of 1-2% in the general population [1]. However, isolated hypoplasia of a single cusp, particularly the right coronary cusp (RCC), is extremely rare. Such anomalies may impair valve coaptation, leading to turbulent flow and subsequent development of progressive stenosis or regurgitation [2]. Owing to its rarity, every reported case adds valuable insight into clinical presentation, diagnosis, and management. We report a case of hypoplastic RCC detected on echocardiography and managed successfully with surgical valve replacement.

## Case presentation

A 13-year-old girl presented with recurrent respiratory tract infection and failure to thrive since her early childhood, and progressive exertional dyspnea (NYHA class II) for six months. She had no significant past medical or family history.

**Figure 1:**
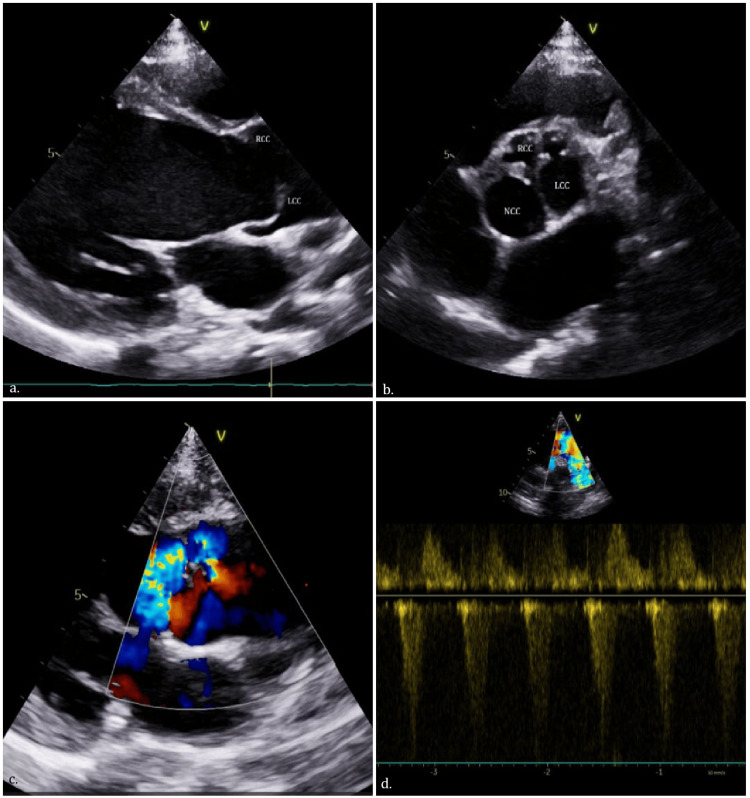
Two-dimensional echocardiography. (a) Parasternal long-axis view showing a hypoplastic RCC and well-formed NCC, with a dilated LV. (b) Parasternal short-axis view demonstrating a trileaflet aortic valve with hypoplastic RCC and well-formed LCC and NCC. (c) Parasternal long-axis view with color Doppler showing a severe posteriorly directed AR jet. (d) Suprasternal view with continuous-wave Doppler of the descending thoracic aorta showing pandiastolic flow reversal, suggestive of severe AR. AR: aortic regurgitation; LV: left ventricle; LCC: left coronary cusp; NCC: non-coronary cusp; RCC: right coronary cusp

**Figure 2:**
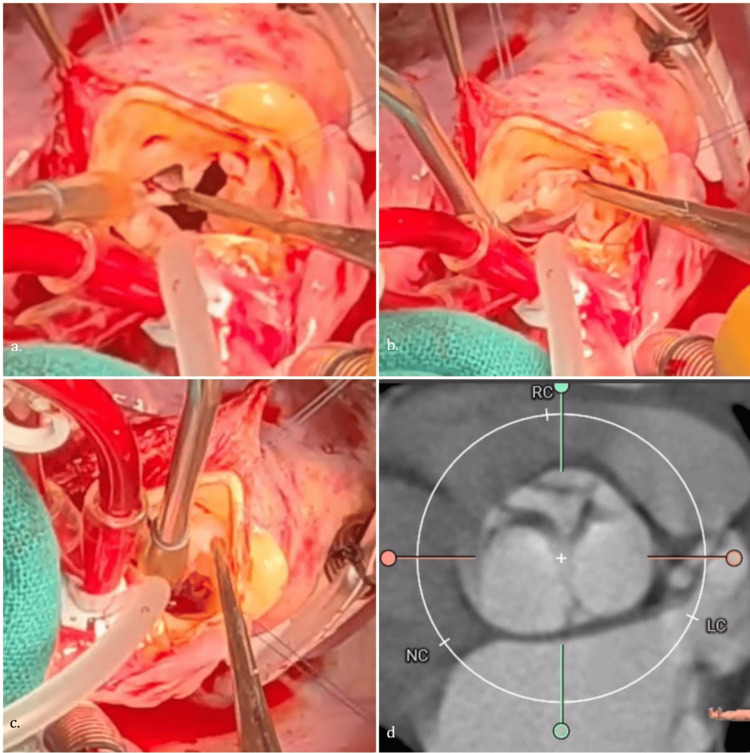
Intra-operative picture showing (a) hypoplastic RCC. The RCC is being held and pulled towards the opposite commissure. It fails to touch other leaflets. (b) The LCC and (c) NCC are normal with normal mobility and could be pulled to touch the opposite commissure. (d) CT aortogram double oblique view showing trileaflet aortic valve with hypoplastic RCC and well-formed LCC and NCC. RCC: right coronary cusp; LCC: left coronary cusp; NCC: non-coronary cusp

On examination, blood pressure was 108/40 mmHg with a regular pulse of 82/min. Her height was 137 cm, and her weight was 28 Kg, with a body mass index (BMI) of 14.9 Kg/m^2^ (underweight). There was cardiomegaly with a hyperdynamic apex. A grade 3/6 ejection systolic murmur was heard over the right second intercostal space radiating to the carotids with an early diastolic murmur at the same site. No signs of heart failure were evident. Electrocardiography showed a normal sinus rhythm, left ventricular enlargement. Chest X-ray revealed cardiomegaly. Transthoracic echocardiography demonstrated a tricuspid aortic valve with a markedly hypoplastic RCC, leading to poor coaptation between the well-formed left and non-coronary cusps and the hypoplastic RCC, causing severe posteriorly directed eccentric jet aortic regurgitation (Figure 1). There was no aortic stenosis. Other valves were normal. The left ventricle (LV) was dilated. End diastolic and end systolic LV dimensions were 56 mm (z score 3.13) and 36 mm (z score 2.76), respectively [3]. Left ventricular ejection fraction was 60%. CT aortography confirmed asymmetric cusp morphology with hypoplastic RCC and normal coronary artery origins (Figure 2d). There was no coronary artery ostial narrowing. The patient underwent aortic valve replacement using a 21 mm Bicarbon SlimlineTM mechanical prosthesis (Sorin Biomedica, Saluggia, Italy). Intraoperative findings confirmed a hypoplastic, rudimentary RCC with thickened, restricted leaflet motion (Figure 2). The postoperative course was uneventful.

## Discussion

Aortic valve anomalies typically manifest as bicuspid morphology or, less frequently, unicuspid or quadricuspid valves. Isolated hypoplasia of a single cusp is rare, with few cases of hypoplastic left coronary cusp presenting with acute myocardial ischemia and left ventricular dysfunction have been reported in the literature [4,5]. The hypoplastic RCC presenting with chronic severe aortic regurgitation, to the best of our knowledge, has not been reported in the literature. Hypoplastic cusps may impair leaflet coaptation, resulting in regurgitation. Over time, these changes result in left ventricular enlargement, heart failure, or predispose to infective endocarditis [6]. Echocardiography is the primary modality, but transesophageal echocardiography, CT, or MRI can provide detailed anatomical confirmation [7]. Treatment depends on the severity of the condition. Asymptomatic patients with preserved function may be followed, while symptomatic patients or those with significant dysfunction require surgical intervention. Valve replacement remains the standard treatment, though repair may be feasible in selected cases [8].

This case highlights the need for a high index of suspicion for congenital cusp anomalies in young patients presenting with unexplained aortic valve disease.

## Conclusions

Hypoplastic RCC of the aortic valve is an exceptionally rare congenital anomaly. Multimodality imaging plays a crucial role in diagnosis. Early surgical intervention is associated with excellent clinical outcomes in symptomatic patients.

## References

[1] Ward C (2000). Clinical significance of the bicuspid aortic valve. Heart.

[2] Nistri S, Sorbo M, Marin M, Palisi M, Scognamiglio R, Thiene G (1999). Aortic root dilatation in young men with normally functioning bicuspid aortic valves. Heart.

[3] Pettersen MD, Du W, Skeens ME, Humes RA (2008). Regression equations for calculation of z scores of cardiac structures in a large cohort of healthy infants, children, and adolescents: an echocardiographic study. J Am Soc Echocardiogr.

[4] Ku L, Ma X (2023). Congenital hypoplastic left coronary cusp. Anatol J Cardiol.

[5] Hayashida K, Okumura S, Kawase T, Kawazoe K (2010). Occlusion of left coronary ostium with a rudimentary aortic cusp. Ann Thorac Surg.

[6] Roberts WC, Ko JM (2005). Frequency by decades of unicuspid, bicuspid, and tricuspid aortic valves in adults having isolated aortic valve replacement for aortic stenosis, with or without associated aortic regurgitation. Circulation.

[7] Detaint D, Michelena HI, Nkomo VT, Vahanian A, Jondeau G, Sarano ME (2014). Aortic dilatation patterns and rates in adults with bicuspid aortic valves: a comparative study with Marfan syndrome and degenerative aortopathy. Heart.

[8] Svensson LG, Blackstone EH, Cosgrove DM 3rd (2003). Surgical options in young adults with aortic valve disease. Curr Probl Cardiol.

